# Altered Spontaneous Brain Activity in Patients with Acute Spinal Cord Injury Revealed by Resting-State Functional MRI

**DOI:** 10.1371/journal.pone.0118816

**Published:** 2015-03-13

**Authors:** Ling Zhu, Guangyao Wu, Xin Zhou, Jielan Li, Zhi Wen, Fuchun Lin

**Affiliations:** 1 Department of Magnetic Resonance Imaging, Zhongnan Hospital of Wuhan University, Wuhan, Hubei, China; 2 Wuhan Center for Magnetic Resonance, State Key Laboratory of Magnetic Resonance and Atomic and Molecular Physics, Wuhan Institute of Physics and Mathematics, Chinese Academy of Sciences, Wuhan, Hubei, China; Beijing Normal University, Beijing, CHINA

## Abstract

**Background:**

Previous neuroimaging studies have provided evidence of structural and functional reorganization of brain in patients with chronic spinal cord injury (SCI). However, it remains unknown whether the spontaneous brain activity changes in acute SCI. In this study, we investigated intrinsic brain activity in acute SCI patients using a regional homogeneity (ReHo) analysis based on resting-state functional magnetic resonance imaging.

**Methods:**

A total of 15 patients with acute SCI and 16 healthy controls participated in the study. The ReHo value was used to evaluate spontaneous brain activity, and voxel-wise comparisons of ReHo were performed to identify brain regions with altered spontaneous brain activity between groups. We also assessed the associations between ReHo and the clinical scores in brain regions showing changed spontaneous brain activity.

**Results:**

Compared with the controls, the acute SCI patients showed decreased ReHo in the bilateral primary motor cortex/primary somatosensory cortex, bilateral supplementary motor area/dorsal lateral prefrontal cortex, right inferior frontal gyrus, bilateral dorsal anterior cingulate cortex and bilateral caudate; and increased ReHo in bilateral precuneus, the left inferior parietal lobe, the left brainstem/hippocampus, the left cingulate motor area, bilateral insula, bilateral thalamus and bilateral cerebellum. The average ReHo values of the left thalamus and right insula were negatively correlated with the international standards for the neurological classification of spinal cord injury motor scores.

**Conclusion:**

Our findings indicate that acute distant neuronal damage has an immediate impact on spontaneous brain activity. In acute SCI patients, the ReHo was prominently altered in brain regions involved in motor execution and cognitive control, default mode network, and which are associated with sensorimotor compensatory reorganization. Abnormal ReHo values in the left thalamus and right insula could serve as potential biomarkers for assessment of neuronal damage and the prediction of clinical outcomes in acute SCI.

## Introduction

Spinal cord injury (SCI) usually leads to a loss of motor and sensory function below the site of injury [1], owing to the disconnection of efferent motor and afferent sensory pathways between the lower body parts and the cortical and subcortical structures [2]. Approximately 50% of patients with SCI are diagnosed with tetraplegia and experience paralysis of all four limbs, whereas the remainder are diagnosed with paraplegia affecting the lower limbs [3]. Cerebral plasticity, the dynamic potential of the brain to reorganize following damage, has been widely explored in the past decade since the development of various neuroimaging and neurophysiological techniques[4].

SCI is known to be associated with widespread structural and functional abnormality in the brain. Patients with SCI have been shown to have lower gray matter volume in the primary motor cortex (M1), primary somatosensory cortex (S1), medial prefrontal cortex and adjacent anterior cingulate cortex (ACC) as well as structural abnormalities in the same areas with reduced gray matter volume, corticospinal and corticopontine tracts [5–8]. PET and fMRI have been used during motor task, revealing increased activation or novel activation of motor areas in both cortical and subcortical areas [9–12]. Almost all previous structural and functional investigations have been conducted in the chronic stage post-SCI. Recently, one anatomical study has detected that atrophic and microstructural changes of corticospinal axons and sensorimotor cortical areas occur within the first month in patients with SCI [13]. To our knowledge, as supported by a literature search, no resting-state functional magnetic resonance imaging (rs-fMRI) study has been performed in patients with acute SCI. Thus, it remains unclear whether the spontaneous brain activity changes in acute SCI patients.

Rs-fMRI based on the blood oxygenation level-dependent (BOLD) technique can detect spontaneous brain activity and endogenous neurophysiological processes of the human brain. Regional homogeneity (ReHo) [14], reflecting the temporal homogeneity of the BOLD signal, is commonly used to detect the spontaneous brain activity [15, 16]. Considering the results of previous studies, it is likely that acute SCI alters spontaneous brain activity in the resting state. Therefore, we first employed ReHo to measure spontaneous brain activity and then performed a voxel-wise analysis to detect brain regions with affected intrinsic brain activity in patients with acute SCI. The associations between spontaneous brain activity and clinical scores were also investigated. Using these approaches, we sought to explore the effects of acute SCI on intrinsic brain activity.

## Materials and Methods

### Subjects

We enrolled 15 patients with acute SCI who were admitted to the Zhongnan Hospital of Wuhan University. Patients with acute SCI satisfied the following inclusion criteria: (i) tetraplegia or paraplegia due to trauma, (ii) acute SCI (within the past 30 days), (iii) right-handedness (assessed using the Edinburgh Handedness Inventory [17]), and (iv) ability to give informed consent. The exclusion criteria as follows: (i) post-traumatic brain injury, (ii) history of seizure, and (iii) contraindications to MRI unless known to be safe in a magnetic environment. A total of 16 gender- and age-matched healthy controls were recruited from the community through local advertisements. They were considered to be healthy, without prior history of neurological illness, and satisfied no exclusion criteria. The data of three SCI patients were excluded because of excessive head motion (see the Data Analysis section). As a result, 12 SCI patients (mean age: 46.67±12.12 years; age range: 28–62 years) and 16 healthy controls (mean age: 46.06±9.44 years; age range: 28–58 years) were included in this study.

The study was approved by the Medical Ethical Committee of the Zhongnan Hospital of Wuhan University (approval number: 2011058). All participants or their relatives provided written informed consent after a complete description of the study was given to them.

### ISNCSCI Assessment

Motor function was assessed using the international standards for the neurological classification of spinal cord injury (ISNCSCI), a revision of the ASIA (American Spinal Injury Association) classification [18]. This assessment provides the level of injury and muscle strength in the key muscles of the upper limbs (C5-C8 and T1 myotomes) and lower limbs (L2-L5 and S1 myotomes). The scales range from 0 to 100, with 100 indicating no impairment and 0 indicating complete impairment.

### Data Acquisition

All subjects were examined using a 3.0 Tesla MRI scanner (Magnetom Trio; Siemens Healthcare, Erlangen, Germany) with an 8-channel phased-array head coil. The rs-fMRI data were acquired as follows: repetition time = 2000 ms, echo time = 30 ms, flip angle = 90°, acquisition matrix = 64×64, field of view = 240×240 mm^2^, and slice thickness = 4.5 mm with no gap. Each brain volume consisted of 30 slices, and each run contained 210 volumes. During the rs-fMRI scanning, subjects were instructed to keep quietly awake with their eyes closed.

### Data Analysis

Data preprocessing was performed using the statistical parametric mapping (SPM8, http://www.fil.ion.ucl.ac.uk/spm). For each subject, the first 10 volumes were discarded to allow for magnetization equilibration and the adaption of the subjects to the circumstance. The remaining volumes were slice-time corrected for the acquisition time and realigned to correct for head motion. Subjects with maximum translation exceeded 3.0 mm or maximum rotation exceeded 3.0° were excluded from this study. Based on this criterion, three SCI patients were excluded from the study. The realigned images were then spatially normalized to the Montreal Neurological Institute space and resampled to 3×3×3 mm^3^. Finally, the linear trend was removed, and a band-pass filter (0.01–0.08 Hz) was applied to reduce the effects of physiological noise.

ReHo analysis was performed using the Resting State fMRI Data Analysis Toolkit (http://restfmri.net/forum/REST). For each voxel, the ReHo value was defined as the Kendall’s coefficient of concordance (KCC) of the time series of this voxel with its nearest 26 neighboring voxels. Each standardized ReHo map was obtained by dividing the raw ReHo map by the global mean ReHo. Finally, the standardized ReHo maps were smoothed using a Gaussian kernel with 6 mm full width at half maximum (FWHM) and were used for the following statistical analysis.

### Statistical Analysis

To determine the brain regions with ReHo values significantly larger than the global mean ReHo, one-sample *t*-test with AlphaSim multiple comparison corrections was performed to obtain the group-specific ReHo map for each group.

The differences of translational and rotational head motion were also assessed between patients and controls. To detect differences in ReHo between groups, a voxel-wise two-sample *t*-test was performed within the whole brain mask. The statistical map was set at a combined threshold of *p*<0.005 for each voxel with a minimum cluster size of 26 voxels (702 mm^3^), resulting in a corrected threshold of *p*_alpha_<0.05 as determined via Monte Carlo simulation (AlphaSim with the following parameters: single-voxel *p* = 0.005, FWHM = 6 mm, and cluster connection radius r = 5 mm, using the whole brain mask). It should be noted that, the standardized ReHo maps rather than the raw ReHo maps were used in the two-sample *t*-test.

Subsequently, the brain regions with altered ReHo compared with healthy controls were extracted as region-of-interest (ROI) masks and these ROI masks were then projected onto the ReHo maps of each subject, and the mean ReHo values within the ROIs were then calculated for post-hoc Pearson correlation analyses. Pearson correlation analyses were performed to detect correlations between the ISNCSCI scores and the mean ReHo values within the brain regions with altered ReHo compared with healthy controls. A *p* value of 0.05 (uncorrected) was used as threshold for significance. Statistical analysis was performed using Statistical Product and Service Solutions Statistics, Version 20.0 for Windows (IBM SPSS Statistics-win64).

## Results

The subjects who participated in this study were all male. No significant difference was found between the two groups in age (*p* = 0.88). The mean post-injury duration was 16.83±4.34 days (range: 9–24 days). There were six paraplegic and six tetraplegic patients. Four patients had suffered complete SCI whereas the remaining eight had suffered incomplete SCI based on the ISNCSCI classification. More detailed information of patients with acute SCI is presented in Table 1. There was no significant difference in head motion between the two groups (two-sample *t*-test, *p* = 0.51 for translational motion and *p* = 0.33 for rotational motion).

**Table 1:** Demographic and clinical information of patients with acute spinal cord injury.

NO	Age at injury (years)	Type of Injury	Time of MRI after injury (days)	AIS	NLI	ISNCSCI motor score
1	62	Fall	24	D	T3	90
2	36	RTA	22	A	T6	50
3	45	Fall	15	B	L2	60
4	66	Fall	15	A	C5	26
5	57	RTA	9	D	C5	66
6	39	Fall	12	A	C4	5
7	28	Fall	16	D	C4	67
8	56	RTA	14	A	T10	50
9	32	Fall	16	A	T9	50
10	47	Fall	21	B	T11	52
11	52	RTA	18	B	C6	55
12	40	RTA	18	B	C4	56

RTA, road traffic accident; AIS, American Spinal Injury Association Impairment Scale; grade A, complete, no motor or sensory function is preserved in the sacral segments S4 and S5; grade B, incomplete, sensory but not motor function is preserved below the neurological level and extends through the sacral segment S4-S5; grade C, incomplete, motor function is preserved below the neurological level, and more than half of key muscles below the neurological level have a muscle grade less than 3; grade D, incomplete, motor function is preserved below the neurological level and at least half of key muscles below the neurological level have a muscle grade of 3 or more; NLI, neurological level of injury; ISNCSCI, international standards for the neurological classification of spinal cord injury.

**Fig 1:**
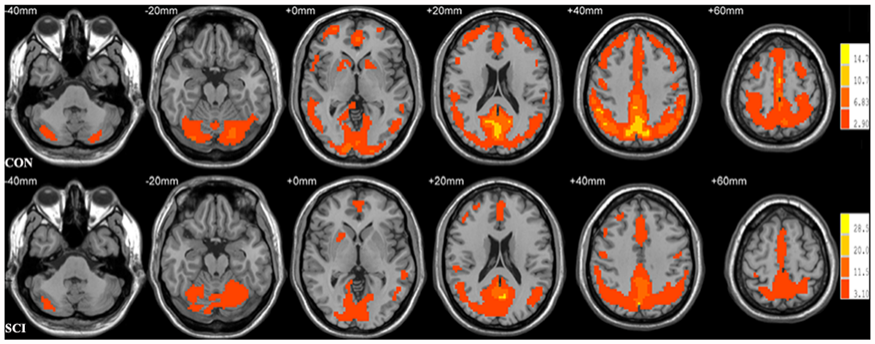
Results of one-sample *t*-test on ReHo maps for healthy controls (CON, uppper) and patients with acute SCI (SCI, lower). Threshold was set to *p*<0.05 with AlphaSim correction. The left side of the image corresponds to the right hemisphere of the brain. The underlying structure image is Ch2 image.

The results of one-sample *t*-test on ReHo maps of the SCI patients and the healthy controls are presented in Fig. 1. Based on visual inspection, the ReHo maps of the two groups appeared to be similar. For both groups, extensive gray matter regions exhibited significantly larger than global mean ReHo values. These regions included the default mode network (DMN, including the precuneus, posterior cingulate cortex, bilateral inferior lateral parietal lobule and medial prefrontal cortex). In addition, we also observed other brain regions to exhibited higher ReHo values, including the visual areas, sensorimotor areas, prefrontal cortex, middle temporal cortex, striatum, thalamus, medial and lateral parietal cortex, cerebellum and execution networks have higher ReHo values.

**Fig 2:**
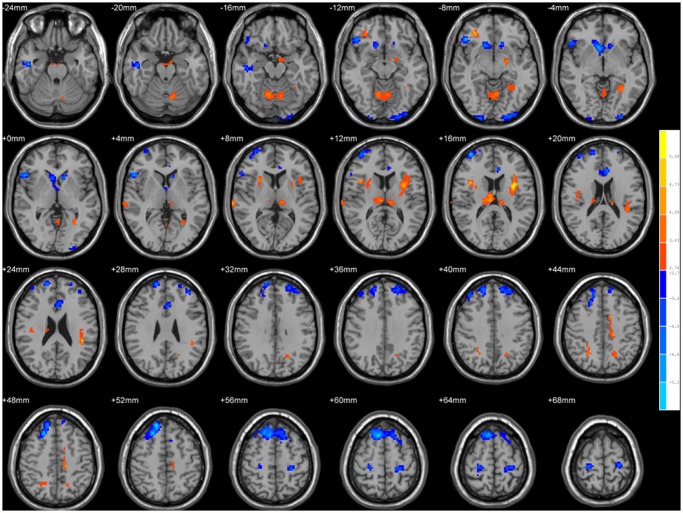
Brain areas with altered ReHo compared with healthy controls (Alphasim corrected, *p*_alpha_<0.05). The blue areas showed decreased ReHo in acute SCI patients relative to healthy controls. The regions are the bilateral primary motor cortex/primary somatosensory cortex, bilateral supplementary motor area/ dorsal lateral prefrontal cortex, right inferior frontal gyrus, bilateral dorsal anterior cingulate cortex and bilateral caudate. The red areas showed increased ReHo in acute SCI patients. The regions include bilateral precuneus, the left inferior parietal lobe, the left brainstem/hippocampus, the left cingulate motor area, bilateral insula, bilateral thalamus and bilateral cerebellum. The left side of the image corresponds to the right hemisphere of the brain. The underlying structure image is Ch2 image.

The group difference in ReHo between the two groups is illustrated in Fig. 2. Compared with the controls, the acute SCI patients showed significantly decreased ReHo in the bilateral precentral/postcentral gyrus (M1/S1), bilateral superior frontal gyrus/supplementary motor area (SMA)/dorsal lateral prefrontal cortex (DLPFC), right inferior frontal gyrus (IFG), bilateral dorsal anterior cingulate cortex (dACC), and bilateral caudate; and increased ReHo in bilateral precuneus, the left inferior parietal lobe (IPL), the left brainstem/hippocampus, the left cingulate motor area (CMA), bilateral insula, bilateral thalamus and bilateral cerebellum (lobe V and VI). The details of the peak coordinates and cluster sizes are listed in Table 2.

**Table 2:** Brain areas with altered ReHo compared with healthy controls (Alphasim corrected, *p*_alpha_<0.05).

Brain areas	Hemisphere	MNI coordinates (cluster maxima, mm)			Peak T values	Cluster size (voxels)
		X	Y	Z		
**SCI>CON**						
Insula	Left	-33	3	15	6.03	90
Insula	Right	39	9	15	4.88	36
Orbital middle frontal gyrus	Right	33	42	-9	4.82	31
Inferior parietal lobule	Left	-42	-36	24	4.76	38
Superior temporal gyrus	Right	72	-24	9	4.65	29
Cingulate motor area	Left	-12	3	45	4.39	44
Cerebellum/ lobe V and VI	Bilateral	12	-63	-15	4.30	166
Thalamus	Right	6	-12	15	4.21	77
Precuneus	Left	-21	-60	30	4.00	29
Thalamus	Left	-9	-24	9	3.96	43
Putamen	Right	27	12	9	3.90	28
Precuneus	Right	24	-54	42	3.86	28
Brainstem/hippocampus	Left	-21	-6	-9	3.81	69
Lingual gyrus	Left	-30	-54	-3	3.79	54
**SCI< CON**						
Superior frontal gyrus/SMA/DLPFC	Bilateral	15	42	51	5.92	783
Inferior frontal gyrus	Right	48	30	-9	5.65	106
Inferior/middle temporal gyrus	Right	48	-12	-24	5.17	73
Caudate	Right	6	15	-3	5.15	110
Caudate	Left	-12	18	0	4.65	35
Precentral/postcentral gyrus/M1/S1	Left	-27	-24	69	4.28	79
Inferior/middle occipital gyrus	Left	-24	-99	-9	4.25	86
Precentral/postcentral gyrus/M1/S1	Right	24	-27	69	4.24	62
Dorsal anterior cingulate cortex	Bilateral	-6	30	18	4.15	70
Inferior/middle occipital gyrus	Right	24	-102	-9	4.14	33
Middle frontal gyrus	Right	39	42	39	3.96	34

MNI: Montreal Neurological Institute; SCI, patients with acute SCI; CON, healthy controls; SMA, supplementary motor area; DLPFC, dorsal lateral prefrontal cortex; M1, primary motor cortex; S1, primary somatosensory cortex.

**Fig 3:**
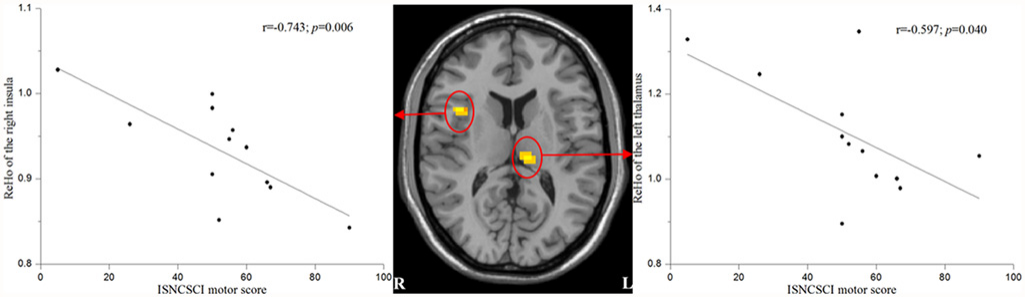
Correlations between the mean ReHo values of the affected regions and ISNCSCI motor scores in patients with acute SCI. Mean ReHo values of the left thalamus and the right insula were negatively correlated with the ISNCSCI motor scores.

We found that the mean ReHo values of the left thalamus and the right insula were negatively correlated with the ISNCSCI motor scores in the SCI patients (r = -0.597, *p* = 0.040 and r = -0.743, *p* = 0.006, respectively) (Fig. 3).

## Discussion

Widespread structural and functional abnormality in the brain has been reported in patients with SCI. In humans, similar to findings from experimental SCI, gray matter becomes atrophic and white matter integrity is reduced [5]. A spinal cord lesion affects primary sensorimotor areas connected to the lesioned area and can result in the reorganization of these and surrounding regions to compensate for sensorimotor loss [19, 20]. However, almost all previous studies have been performed in the chronic stage post-SCI. In the current study, ReHo was first employed to explore the changes in spontaneous brain activity and a voxel-wise analysis was then performed to detect brain regions with altered intrinsic brain activity in acute SCI patients. The results indeed demonstrated that acute distant neuronal damage has an immediate impact on spontaneous brain activity.

ReHo measures the similarity or coherence of low frequency fluctuations (LFFs) within a given area based on hemodynamics. The LFF BOLD signal has been suggested to reflect spontaneous neuronal activity [21–23]. Therefore, altered ReHo is most likely relevant to changes in the temporal aspects of regional spontaneous neural activity. Higher ReHo is thought to indicate greater temporal synchrony, whereas lower values are thought to represent decreased local coherence [24]. Results of one-sample *t*-test on ReHo maps of the two groups observed in the present study were consistent with previous studies [25–27], whereas abnormal ReHo was observed in cortical and subcortical brain regions in patients with acute SCI.

Compared with the controls, the acute SCI patients showed decreased ReHo prominently in brain regions involved in motor execution and cognitive control. First, brain regions with significantly decreased ReHo included the bilateral M1, SMA, right IFG and bilateral S1. M1, SMA and IFG are known to be critical in motor execution. The M1 is not only an executive motor area but also an area that contributes to movement sequence preparation [28] and motor control [29]. The SMA is thought to play a role in higher order activities related to movement, such as selection, preparation and sequencing of movements, as well as in movement execution [30]. The IFG programmes the sequential ordering of motor execution and is especially active in motor tasks of great difficulty or tasks which demand on selective attention [31]. Because sensorimotor function comprising motor function and sensory feedback from the spinal cord to the brain are expected to be greatly impaired or even absent in patients with acute SCI, the decreased ReHo observed in the M1, SMA, IFG and S1 might reflect the motor execution deficits and a state of sensory deafferentation of these patients. Voxel-based morphometry (VBM) studies have found SCI patients with reduced gray matter volume in M1 and S1 [5, 7, 8], which might be the structural basis of the altered spontaneous brain activity in patients with acute SCI. In addition, diffusion tensor imaging (DTI) has been used to evaluate the white matter microstructural changes following SCI. DTI analysis revealed structural abnormalities in the brain regions with reduced gray matter volume as well as the corticospinal and corticopontine tracts of SCI subjects [5, 6]. A rest SPECT study has also revealed regional blood flow reduction in M1, SMA, other movement-cortical areas and S1 in patients with SCI [32]. The decreased ReHo in S1 observed in our study is in line with neurophysiological evidence obtained from animal experiments: immediately (within minutes) after thoracic transection of the spinal cord, the S1 cortical spontaneous activity at rest becomes strikingly slower and overall more silent [33].

Moreover, the patients with acute SCI showed decreased ReHo in other brain regions associated with cognitive control, i.e., the bilateral dACC, DLPFC and caudate. Cognitive control supports flexible behavior by selecting actions that are consistent with our goals and appropriate to our environment [34]. There is ample evidence that the control of any voluntary movement relies upon both higher-level cognitive and lower-level movement mechanisms [35]. Studies using functional neuroimaging techniques have related cognitive control to activity in the ACC and DLPFC [36]. Various functions have been ascribed to the dACC, including the modulation of attention or executive functions, complex motor control and the anticipation of cognitively demanding tasks [37]. Changes in DLPFC activity are often associated with the modulation of ACC activity and can be explained using several computational models that define the lateral PFC, ACC and parietal cortex as the core components involved in executive control [38]. The caudate is consider to be involved in cognitive functions [39] playing a critical role in supporting the planning and execution of strategies and behavior required for complex goals [40]. The decreased spontaneous brain activity observed in regions within the cognitive control network may indicate a reduced level of cognitive control capability in patients with acute SCI, which may be an additional cause for the hypoactivation of the motor execution network.

Precuneus, IPL and hippocampus are components of DMN [41]. The increased ReHo in these brain regions might suggest that the DMN is abnormal in patients with acute SCI. The other brain regions with increased ReHo in the acute SCI patients included the left CMA, bilateral insula, bilateral thalamus and cerebellum, may be associated with the sensorimotor compensatory reorganization related to both motor execution deficits and sensory deafferentation. The CMA has been suggested to play a pivotal role in processing the information necessary to select voluntary actions in accordance with the subject’s internal and external requirements [42]. The human insula has been revealed map to the sensorimotor network of the brain. This area has been repeatedly demonstrated to be involved in various somato- and viscerosensory stimuli. In addition to this sensory processing, movement was elicited by electrical stimulation of this region in humans, which indicates that this region plays a role in sensorimotor processing [43]. The thalamus is a relay center subserving both sensory and motor mechanisms [44]. Clinical studies suggest that cerebellum lobes V and VI are principally engaged in motor control and somatosensory functions [45]. Strong additional activation of the thalamus and cerebellum has been shown using PET in patients with SCI during the performance of motor task [10]. The authors of this study assumed that when afferent input from the spinal cord is reduced, more complex processing of the remaining inputs leads to stronger activation, or possibly to disinhibition, of the neuronal centers involved, i.e., the thalamus and cerebellum.

Furthermore, the altered spontaneous brain activity of the left thalamus and right insula may reflect clinical outcomes. We found negative correlations between the ReHo values of the left thalamus and right insula and the ISNCSCI motor scores in the patients with acute SCI. As mentioned above, both the insula and thalamus play critical roles in integrating sensorimotor processing. The explanation of these negative correlations might be that more severe motor impairment leads to more evident compensatory reorganization. However, it is difficult to explain why there is a lateralization in these two brain regions.

Several limitations of our study should be mentioned. Because of the relatively low incidence, acuteness and severity of this disorder, only 12 male patients in the acute stage were included in the present study. Studies have demonstrated that the post-injury brain reorganization may follow a dynamic time course [46, 47]. Thus, another limitation was that we did not conduct this work as a longitudinal study because of low compliance. Finally, further analysis of function connectivity of the involved brain regions in the resting state should be conducted in future work.

In conclusion, the abnormal ReHo observed after remote spinal lesions demonstrated that even acute distant neuronal damage has an immediate impact on spontaneous brain activity. The spontaneous brain activity in brain regions associated with sensorimotor, cognitive control and DMN have changed in patients with acute SCI. Abnormal ReHo values in the left thalamus and right insula could serve as potential biomarkers for the assessment of the neuronal damage and the prediction of clinical outcomes in acute SCI.
